# Supplementary material for: Full-length single-cell BCR sequencing paired with RNA sequencing reveals convergent responses to pneumococcal vaccination
Source: Commun Biol. 2024 Sep 28;7:1208. doi: 10.1038/s42003-024-06823-0 (PMC11438910; doi:10.1038/s42003-024-06823-0)
Supplement: Supplementary file 3 — Description of Additional Supplementary Files [file 42003_2024_6823_MOESM3_ESM.pdf]

Description of Additional Supplementary Files

File name: Supplementary Data 1

Description: The source data behind Figure 6h.

File name: Supplementary Data 2

Description: The source data behind Figure 8.

File name: Supplementary Data 3

Description: Primer oligo sequences used to construct and sequence B3E-seq libraries.

File name: Supplementary Data 4

Description: Primer oligo sequences used to construct and sequence bulk BCR sequencing libraries.
