# Supplementary material for: Full-length single-cell BCR sequencing paired with RNA sequencing reveals convergent responses to pneumococcal vaccination
Source: Commun Biol. 2024 Sep 28;7:1208. doi: 10.1038/s42003-024-06823-0 (PMC11438910; doi:10.1038/s42003-024-06823-0)
Supplement: Supplementary file 8 — Reporting Summary [file 42003_2024_6823_MOESM8_ESM.pdf]

nature portfolio

Corresponding author(s): COMMSBIO-23-2042B

Last updated by author(s): July 1, 2024

Reporting Summary

Nature Portfolio wishes to improve the reproducibility of the work that we publish. This form provides structure for consistency and transparency in reporting. For further information on Nature Portfolio policies, see our Editorial Policies and the Editorial Policy Checklist.

Statistics

For all statistical analyses, confirm that the following items are present in the figure legend, table legend, main text, or Methods section.

n/a	Confirmed	
☐	☒	The exact sample size (*n*) for each experimental group/condition, given as a discrete number and unit of measurement
☐	☒	A statement on whether measurements were taken from distinct samples or whether the same sample was measured repeatedly
☐	☒	The statistical test(s) used AND whether they are one- or two-sided<br>*Only common tests should be described solely by name; describe more complex techniques in the Methods section.*
☒	☐	A description of all covariates tested
☐	☒	A description of any assumptions or corrections, such as tests of normality and adjustment for multiple comparisons
☐	☒	A full description of the statistical parameters including central tendency (e.g. means) or other basic estimates (e.g. regression coefficient) AND variation (e.g. standard deviation) or associated estimates of uncertainty (e.g. confidence intervals)
☐	☒	For null hypothesis testing, the test statistic (e.g. *F*, *t*, *r*) with confidence intervals, effect sizes, degrees of freedom and *P* value noted<br>*Give P values as exact values whenever suitable.*
☒	☐	For Bayesian analysis, information on the choice of priors and Markov chain Monte Carlo settings
☒	☐	For hierarchical and complex designs, identification of the appropriate level for tests and full reporting of outcomes
☐	☒	Estimates of effect sizes (e.g. Cohen's *d*, Pearson's *r*), indicating how they were calculated

Our web collection on statistics for biologists contains articles on many of the points above.

Software and code

Policy information about availability of computer code

Data collection	Python v3.6.4, pRESTO v0.5.6, Change-O v0.4.6, IgBLAST v1.14.0, R v4.1.1, Seurat v4.1.1, FlowJo v10.8.1, FastxToolKit v0.0.13
Data analysis	ggplot2 v3.5.0, dplyr v1.1.4, pheatmap v1.0.12, rcolorbrewer v1.1.3, ggrastr v1.0.2, ggbeeswarm v0.7.2, stringr v1.5.1, reshape2 v1.4.4

For manuscripts utilizing custom algorithms or software that are central to the research but not yet described in published literature, software must be made available to editors and reviewers. We strongly encourage code deposition in a community repository (e.g. GitHub). See the Nature Portfolio guidelines for submitting code & software for further information.

Data

Policy information about availability of data

All manuscripts must include a data availability statement. This statement should provide the following information, where applicable:

- Accession codes, unique identifiers, or web links for publicly available datasets
- A description of any restrictions on data availability
- For clinical datasets or third party data, please ensure that the statement adheres to our policy

Raw and processed data from this study has been deposited on GEO with accession numbers GSE232873 and GSE266697.

Human research participants

Policy information about studies involving human research participants and Sex and Gender in Research.

Reporting on sex and gender	Not applicable
Population characteristics	Not applicable
Recruitment	Not applicable
Ethics oversight	Not applicable

Note that full information on the approval of the study protocol must also be provided in the manuscript.

Field-specific reporting

Please select the one below that is the best fit for your research. If you are not sure, read the appropriate sections before making your selection.

☒ Life sciences ☐ Behavioural & social sciences ☐ Ecological, evolutionary & environmental sciences

For a reference copy of the document with all sections, see nature.com/documents/nr-reporting-summary-flat.pdf

Life sciences study design

All studies must disclose on these points even when the disclosure is negative.

Sample size	As our study was exploratory, the sample size was pre-determined.
Data exclusions	Data exclusions are described in the Methods. In brief, cells were pre-filtered during alignment based on number of transcripts detected and quality of gene expression.
Replication	Reproducibility of our method was performed by processing technical replicates of experimental samples, which resulted in similar data.
Randomization	Samples from Rhesus macaques were not selected randomly but were selected to encompass a range of responsiveness to the glycoconjugate vaccines studied here. As the study was exploratory and did not attempt to establish correlations to vaccine responsiveness, no randomization is required
Blinding	Blinding was not necessary, as no correlations to outcomes were studies.

Reporting for specific materials, systems and methods

We require information from authors about some types of materials, experimental systems and methods used in many studies. Here, indicate whether each material, system or method listed is relevant to your study. If you are not sure if a list item applies to your research, read the appropriate section before selecting a response.

Materials & experimental systems

n/a	Involved in the study	
☐	☒	Antibodies
☐	☒	Eukaryotic cell lines
☒	☐	Palaeontology and archaeology
☐	☒	Animals and other organisms
☒	☐	Clinical data
☒	☐	Dual use research of concern

Methods

n/a	Involved in the study	
☒	☐	ChIP-seq
☐	☒	Flow cytometry
☒	☐	MRI-based neuroimaging

Antibodies

Antibodies used	BV60S-conjugated anti-lgM (MHM-88, Biollegend)<br>BV785-conjugated anti-CD20 (2H7; Biolegend)<br>Dylight 405-congjuatated anti-CD19 (CB19; R&D systems)<br>VS00-conjugated CD3 (SP34-2, BD Biosciences)

	PE-CyS-conjugated anti-lgG (G18-145, BD Biosciences)<br>Total-seq A anti-human hashtag antibody (Biolegend)
Validation	All antibodies used undergo validation by their manufacturer.

Eukaryotic cell lines

Policy information about cell lines and Sex and Gender in Research

Cell line source(s)	HEK 293T cells were purchased from ATCC (CRL-3216).
Authentication	HEK 293T cells were validated by the manufacturer. We did not perform additional authentication.
Mycoplasma contamination	HEK 293T cells were delivered mycoplasma-free. After thawing, cells were used immediately for transfection, as described, and were not kept in prolonged culture. We did not perform additional mycoplasma testing.
Commonly misidentified lines<br>(See ICLAC register)	No commonly misidentified lines were used

Animals and other research organisms

Policy information about studies involving animals; ARRIVE guidelines recommended for reporting animal research, and Sex and Gender in Research

Laboratory animals	Infant rhesus macaques (age 3-6 months at the start of the study) were housed with their moms for the duration of the study at the New Iberia Research Center (NIRC), University of Louisiana. Control serum and PBMC samples were collected from adult Rhesus Macaques at NIRC from na"ive animals which have not been previously vaccinated with pneumococcal vaccines.
Wild animals	No wild animals were used.
Reporting on sex	Age- and sex-matched infant rhesus macaques were randomly divided into groups for vaccination.
Field-collected samples	This study did not involve field-collected samples.
Ethics oversight	The infant Rhesus macaque experimental protocol was approved by IACUC at both Pfizer Inc and NIRC.

Note that full information on the approval of the study protocol must also be provided in the manuscript.

Flow Cytometry

Plots

Confirm that:

☒ The axis labels state the marker and fluorochrome used (e.g. CD4-FITC).

☒ The axis scales are clearly visible. Include numbers along axes only for bottom left plot of group (a 'group' is an analysis of identical markers).

☒ All plots are contour plots with outliers or pseudocolor plots.

☒ A numerical value for number of cells or percentage (with statistics) is provided.

Methodology

Sample preparation	Cryopreserved PBMC were thawed in AIM-V media and washed twice with PBS. Tetramer reagent was prepared by combining equal volumes of streptavidin-PE or streptavidin-APC solutions (both from BD) at 200 ug/ml with polysaccharide-biotin solution at 20 ug/ul. Cells were then stained with eBioscience Fixable Viablity Dye eFlour 780 (ThermoFisher) and Fc block (BD) on ice for 30 minutes. Cells were then washed twice with PBS and then stained with 25 ul of each ST3-PE tetramer and ST3-APC tetramer on ice for 30 minutes. Cells were then washed three times in FBS staining buffer (BD) and were then stained with BV605-conjugated anti-lgM (MHM-88), BV786-conjugated anti-CD20 (both from Biolegend), Dylight 405-congjuatated anti-CD19 (CB19) (R&D systems), VS00-conjugated CD3 (SP34-2), PE-CyS-conjugated anti-lgG (G18-145) (both from BD), and Total-seq A anti-human hashtag antibody (Biolegend) on ice for 30 minutes. Live+CD3-CD19+CD20+ST3+ and Live+CD3-CD19+CD20+ST3- cells were sorted on a BD Aria 3 sorter, and data was analyzed using Flowjo v10.
Instrument	Samples were sorted on a FACSAria Ill instrument (BD Biosciences).
Software	Data was analyzed using FlowJo vl0.8.1 (BD Biosciences).
Cell population abundance	Purity of the samples was determined by correlating gene expression results in RNA-Seq to antibody targets in flow cytometry. Minimal number of non-B cells were detected.

Gating strategy	Complete gating strategy is illustrated in Supplemental Figure 3.

☒ Tick this box to confirm that a figure exemplifying the gating strategy is provided in the Supplementary Information.
